# Comprehensive molecular epidemiology of *Acinetobacter baumannii* from diverse sources in Nigeria

**DOI:** 10.1186/s12866-025-03917-5

**Published:** 2025-03-31

**Authors:** Samuel O. Ajoseh, Abdul-Azeez A. Anjorin, Wasiu O. Salami, Hanka Brangsch, Heinrich Neubauer, Gamal Wareth, Kabiru O. Akinyemi

**Affiliations:** 1 Department of Microbiology, Faculty of Science, Lagos State University, P.M.B 0001, Ojo, Lagos, Nigeria; 2 Institute of Bacterial Infections and Zoonoses, Friedrich-Loeffler-Institut (FLI), 07743 Jena, Germany

**Keywords:** *Acinetobacter baumannii*, Whole genome sequencing, Multilocus sequence analysis, MDR genes, Virulence genes, Molecular epidemiology, Nigeria

## Abstract

**Background:**

*Acinetobacter baumannii*, a Gram-negative bacterium, is a public health threat due to its role in nosocomial infections and increasing antibiotic resistance. In Nigeria, data on the molecular epidemiology of *A. baumannii* is scarce. This study investigates the genetic diversity and the presence of antimicrobial resistance determinants and virulence-related genes in whole-genome sequencing data of 189 Nigerian *A. baumannii* isolates deposited in public repositories. Genotypes were determined in-silico by multilocus sequence typing (MLST) and core genome MLST (cgMLST). Further, antimicrobial resistance (AMR) and virulence-related genes were analyzed.

**Results:**

Most isolates (57.67%) originated from South-west Nigeria. Isolates of human origin accounted for 33.86%, while environmental sources comprised 6.87%, and 59.27% lacked information on the source of isolation. The cgMLST analysis revealed a multitude of genomic lineages circulating in Nigeria. The MLST Oxford scheme identified 44 sequence types (STs) in 62.96% of strains, with ST1089 being the most prevalent. The MLST Pasteur could assign 95.77% of strains to 49 STs, with ST2(IC2) and ST85(IC9) being the most dominant. Antimicrobial resistance analysis detected 168 genes encoding resistance to 12 antibiotic classes, with cephalosporin, carbapenem, and aminoglycoside resistance genes being the most prevalent. Notably, *bla*_ADC−79_ (23.81%), *bla*_OXA−23_ (30.69%), and *aph*(3″)-Ib (30%) were frequent variants encountered. Seventeen multi-efflux system genes conferring resistance to multiple antibiotic classes were identified. Virulence gene analysis revealed 137 genes encoding six mechanisms, with genes for nutritional factors, effector delivery systems, and biofilm production being the most prevalent.

**Conclusion:**

This study highlights the diversity in AMR and virulence genes of *A. baumannii* in Nigeria, emphasizing the need for ongoing genomic surveillance to inform infection control and develop antibiotic resistance management strategies.

**Supplementary Information:**

The online version contains supplementary material available at 10.1186/s12866-025-03917-5.

## Background

*Acinetobacter baumannii* (*A. baumannii*) is a Gram-negative, non-flagellated bacterium that has emerged as a notorious opportunistic pathogen, particularly in hospital settings [1]. *A. baumannii* is a One-Health pathogen, as it was isolated from humans, animals, and foods, as well as from environmental sources [2–5]. It has been classified by the World Health Organization (WHO) as one of the most critical multidrug-resistant (MDR) organisms requiring the development of novel antibiotics [6]. This bacterium is a member of the ESKAPE (*Enterococcus faecium *,* Staphylococcus aureus*,* Klebsiella pneumoniae*,* Acinetobacter baumannii*,* Pseudomonas aeruginosa*, and *Enterobacter* species) group, a collection of bacterial pathogens known for their high rates of multidrug and extensive drug resistance (MDR and XDR), making them a major cause of nosocomial infections worldwide [6]. *A. baumannii* possesses intrinsic resistance mechanisms, including reduced membrane permeability, efflux pump activity, and the production of diverse β-lactamases enzymes [7]. However, resistance in this pathogen is often associated with mobile genetic elements (MGEs), which facilitate the rapid dissemination and maintenance of resistance genes between bacterial species [7, 8]. *A. baumannii* can also acquire resistance through mutational changes in its chromosomal structure, horizontal gene transfer [9], and naturally occurring intrinsic resistance genes [7]. Beyond its intrinsic resistance encoded by a large resistance island within its genome, *A. baumannii* can rapidly acquire additional extrinsic resistance through cross-species horizontal gene transfer during antibiotic therapy [8]. The acquisition of antimicrobial resistance (AMR) genes has been linked to a chromosome and various plasmids [6] and has been reported to be potential contributing factors for pathogenesis in *A. baumannii* [8, 10, 11].

In Nigeria, *A. baumannii* has been documented in hospital and community settings [12], and its genetic diversity highlights the complexity of its epidemiology based on recent studies on circulating clones of *A. baumannii* in Nigerian hospitals, where some strains were found to exhibit resistance to carbapenem antibiotics [13, 14]. Furthermore, a high incidence of carbapenem-resistant *A. baumannii* with *bla*_NDM−1_ and *bla*_OXA−23_ resistance genes was reported in hospital wastewaters in Ibadan, Nigeria [15]. Despite these reported cases of *A. baumannii* from clinical and environmental sources in Nigeria, the molecular epidemiology of antibiotic-resistant *A. baumannii* strains originating from diverse sources remains poorly understood [12, 16], although it is crucial for developing effective infection control strategies and antibiotic stewardship programs. The outbreak investigations of *A. baumannii* have traditionally relied on pulsed-field gel electrophoresis (PFGE). Still, its limitations in terms of reproducibility and portability have become increasingly evident in the typing of this bacterium [17]. However, multilocus sequence typing (MLST) offers a relatively straightforward approach, although its resolution is insufficient for high-fidelity outbreak tracking [18]. Furthermore, core genome multilocus sequence typing (cgMLST) has emerged as a powerful tool for the epidemiological study of *A. baumannii* globally [17]. This method offers high discriminatory power and accuracy in identifying outbreak strains, facilitating the tracking of transmission pathways, and implementing targeted interventions [17]. The advent of affordable whole-genome sequencing (WGS) has revolutionized the field of molecular epidemiology, enabling high-resolution typing and comprehensive genomic comparisons instead of a few loci [19]. Moreover, WGS facilitates the detection of both intrinsic and acquired antimicrobial resistance determinants, thereby enhancing our understanding of the circulating resistomes [17]. Molecular epidemiological studies have identified nine major international clones (IC1-9) of *A. baumannii*, with IC2 being the most prevalent and frequently associated with the acquired carbapenemase OXA-23 globally [17, 20, 21]. A study encompassing isolates from 47 countries, including three African nations - Egypt, South Africa, and Morocco - revealed a predilection for IC2, followed by IC5 and IC9 [21]. Furthermore, a study conducted in Sudan corroborated these findings, identifying a pronounced prevalence of international clone 2 (IC2) among carbapenemase-resistant *A. baumannii* (CRAB) isolates, which were notably characterized by the presence of OXA-66 and OXA-23 carbapenemase genes [17]. However, there exists a significant knowledge gap on the epidemiological genomic profiles of *A. baumannii* in Nigeria and other low-and middle-income countries (LMICs), primarily due to limited research infrastructure and resources. In light of the transnational nature of antimicrobial resistance, it is imperative to generate epidemiological and genomic data to assess the burden of this infectious pathogen and track its antimicrobial resistance in Nigeria. Hence, this study used information from 189 genomes of Nigerian *A. baumannii* strains deposited in public repositories between 2015 and 2024 to provide molecular epidemiological insights into the circulating strains in Nigeria, their virulence, and antimicrobial genomic determinants.

## Materials and methods

### Data collection and quality control

Sequencing data of *A. baumannii* from Nigeria was retrieved from public repositories GenBank databases, including the National Center for Biotechnology Information (NCBI), European Nucleotide Archive (ENA), and the DNA Data Bank of Japan (DDBJ), (Accessed on 31.08.2024).

Depending on the type of data (assemblies or raw read data), different quality control measures were taken. Quality control of raw read data was done using FastQC v0.11.7 (https://www.bioinformatics.babraham.ac.uk/projects/fastqc/). Intraspecific contamination was detected via ConFindr v0.8.1 [22], while interspecific contamination was detected using Kraken2 v2.0.7_beta software [23]. The genomes were assembled using Shovill v1.0.4 (https://github.com/tseemann/shovill) with SPAdes. These *de novo* assemblies and the assemblies downloaded from GenBank were checked for completeness and contamination with CheckM v1.2.3 [24]. General assembly statistics were calculated using QUAST v5.2.0 [25]. Furthermore, the species identity of the contigs was checked using Kraken2 v2.0.7_beta. Contigs that were identified as being different from *A. baumannii* by Kraken2 were checked manually to see whether these contigs were plasmid-borne or contaminant by using a BLAST online search [26]. All data verified as not pure *A. baumannii* were excluded from data analysis.

### Analysis of retrieved data

All analyses were done using the downloaded assemblies or assembled Short Read Archive (SRA) data, which were of sufficient quality. Core genome multilocus sequence typing (cgMLST) was carried out using Ridom Seqsphere + v8.2.0(Ridom GmbH, Münster, Germany) using the default parameter threshold 9, with the scheme developed by Higgins et al. [18]. MLST was done via mlst v2.23.0 (https://github.com/tseemann/mlst) with Oxford and Pasteur schemes as provided by PubMLST [27–29]. Antimicrobial resistance genes were detected in the *A. baumannii* genomes using ABRicate v1.0.1 (https://github.com/tseemann/abricate) with the CARD [30], Resfinder [31] and NCBI [32] databases. Furthermore, AMRFinderplus v3.11.26 with the -O option for *A. baumannii*, which allows the detection of point mutations that potentially confer antibiotic resistance, was used. Virulence genes were detected by screening using ABRicate with the included Virulence Factor Database (vfdb) [33]. The coverage and percentage of identity for detecting the resistance and virulence genes are 100 and 96%, respectively.

## Results

### Data analysis

**Fig. 1:**
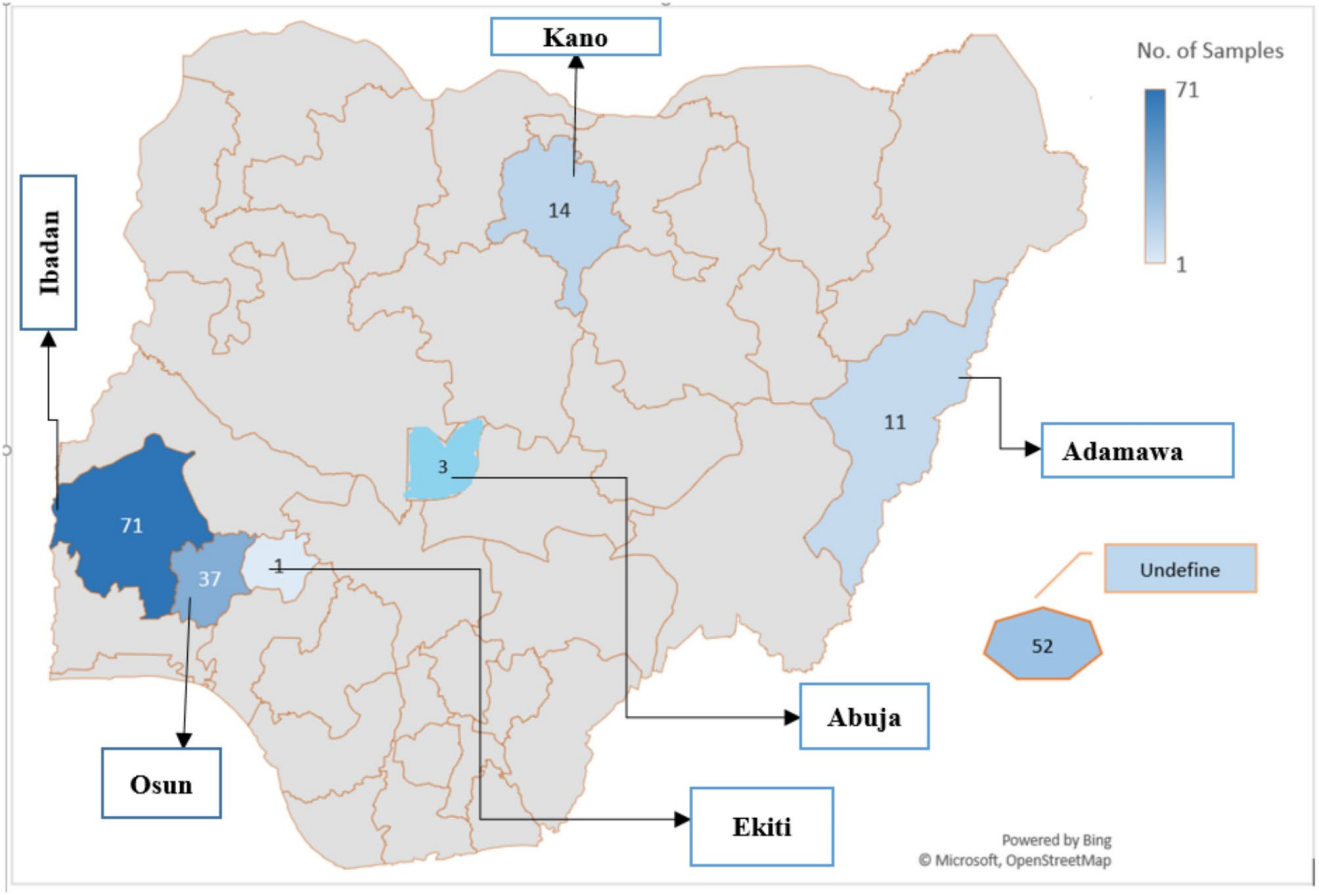
Geographical origin of deposited *A. baumannii* WGS data from Nigeria (2015–2024). Numbers and coloration indicate the number of available database entries

A total of 189 genomes of Nigerian *A. baumannii* were retrieved from NCBI (*n* = 28) and ENA (*n* = 161). No data has been found at the DNA Data Bank of Japan (DDBJ). Starting in 2015, the majority of these data were deposited in 2020 and 2021, with 64 entries submitted each year. The last submission was a single entry in 2023, as of August 31, 2024. Geographically, the majority of the 189 samples (109 sequences, accounting for 57.67%) originated from South-west Nigeria, predominantly from Oyo State (*n* = 71), followed by Osun State (*n* = 37), and a single entry from Ekiti State. From Northern Nigeria, 15.35% (29 sequences) were uploaded, with the highest number from Kano State (*n* = 14), followed by Adamawa (*n* = 11) and a few from Abuja (*n* = 3). Notably, there were no samples from the South-South and Southeast regions of Nigeria. Additionally, 52 sequences (26.98%) lacked specific geographic information beyond the country of origin (Fig. 1).

The majority of entries, 112 sequences (59.27%), lacked information on the source of isolation. A third of the samples (64 sequences, 33.86%) were derived from human sources, and 13 sequences (6.87%) were from environmental sources. Among the 64 *A. baumannii* entries from human sources, 34 were obtained from rectal swabs, 16 from blood samples, five from wound cultures, four from urine, and two from unspecified human sample sources. The least represented sources included pleural aspirates, sputum, and stool, with one sequence each. Regarding the 13 environmental isolates, four were recovered from school toilet floors, while two each were found in wastewater and hospital surfaces. Other environmental sources with a singular sequence include soil, an unpolluted field, a hospital bed, a door handle, and a hospital drawer.

### MLST analysis

The multilocus sequence typing (MLST) analysis based on the Pasteur scheme assigned 181 out of 189 strains into 49 distinct sequence types (STs) (Fig. 1). The remaining eight strains could not be assigned to an existing ST and comprised three isolates from unknown sources, three rectal swab isolates, one urine isolate, and one hospital surface isolate. ST2 was the most dominant and diverse with regard to sample type, occurring 22 times (11.64%), followed by ST85, which occurred 15 times (7.94%). The geographical distribution of Pasteur sequence types (STs) revealed distinct patterns across different regions in Nigeria. In Adamawa state, Northeast region, three STs were identified, with ST2 belonging to international clone 2 (IC2) being the most prevalent, accounting for six isolates, followed by ST52 (*n* = 3) and ST85 (*n* = 2), which belongs to IC9. In Ibadan, Oyo state, South-west region, 28 different ST types were identified, and ST32, classified as IC3, was the most frequent, occurring in eight *A. baumannii* isolates followed by ST409 (*n* = 7), ST2243 (*n* = 6), and ST10 (*n* = 5) belonging to IC2. However, one isolate of ST1 of IC1 was also identified in this state/location. In Osun state, another South-west region, 14 distinct STs were identified, with ST2 of IC2 being the most prevalent, occurring in 10 *A. baumannii* isolates, followed by ST85 belonging to IC9 (*n* = 8) and ST149 (*n* = 3). Five distinct STs were documented in Kano state, northwest Nigeria, with ST267 emerging as the most dominant strain. However, two *A. baumannii* isolates (ST2 and ST10) belonging to IC2 were identified in this region. Notably among isolates with unknown locations within Nigeria. A total of 22 distinct STs were recorded from *A. baumannii* isolates recovered from unspecified locations in Nigeria, with ST1 belonging to IC1 being the most frequent, occurring in six isolates, followed by ST2 of IC2 (*n* = 5), ST25 (*n* = 5), and ST85 (*n* = 5) that is classified as IC9.

**Fig. 2:**
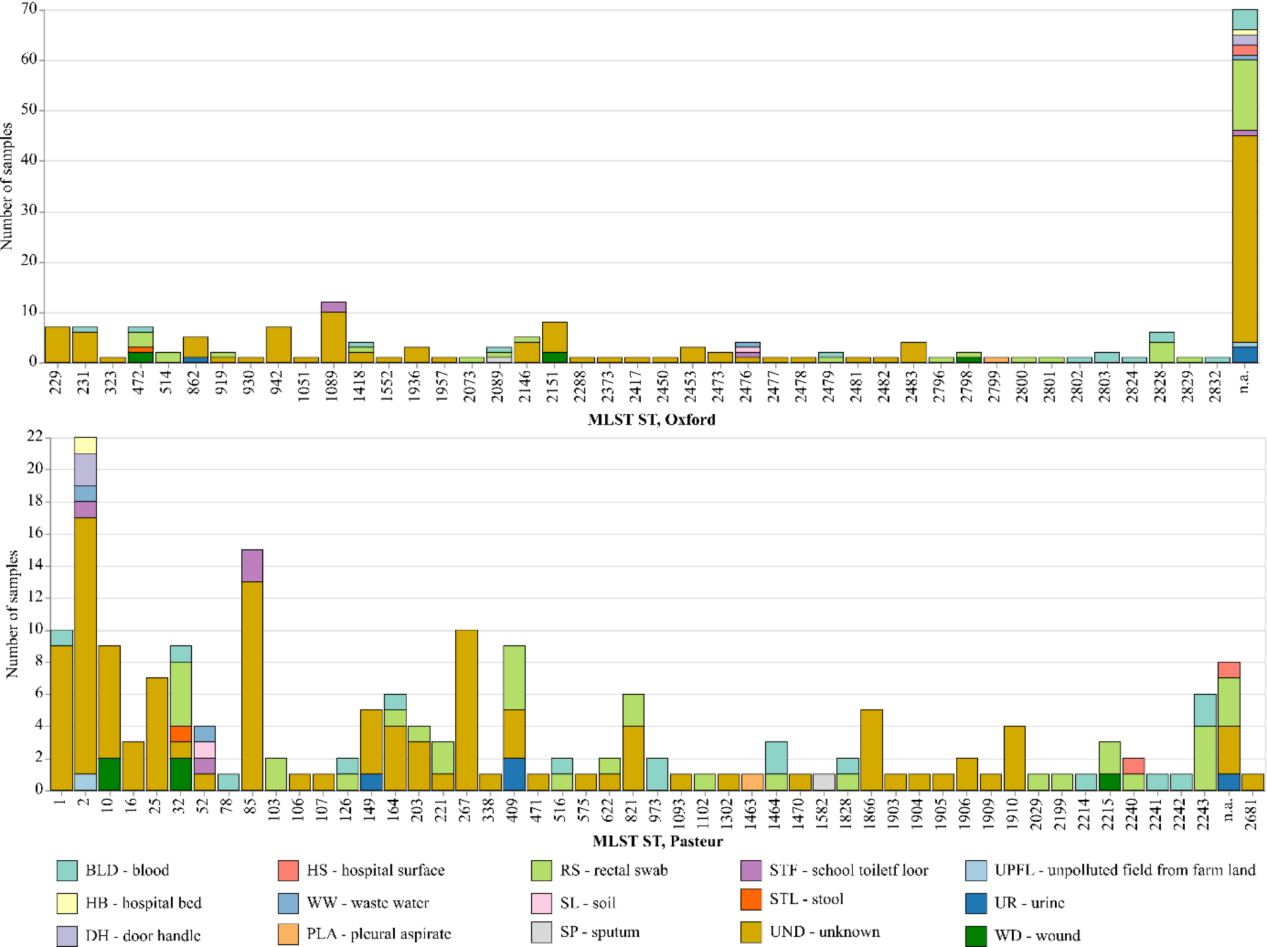
The number of samples per MLST ST, identified by the Pasteur and Oxford schemes, respectively, was colored according to the sample source

In contrast to the Pasteur scheme, the analysis utilizing the Oxford MLST scheme resulted in the detection of 44 distinct sequence types in 119 strains (62.96%), whereas for 70 strains (37.03%), no defined ST could be found (Fig. 2). Notably, among the typed strains, ST1089 emerged as the most frequent, accounting for 12 isolates (6.35%) derived from school toilet floors (*n* = 2) and undefined sources (*n* = 10). The second most prevalent ST was ST2151, which was detected in eight isolates (4.23%). Interestingly, the eight strains that were not assigned to an ST by the Pasteur scheme were also of unknown ST in the Oxford scheme. The geographic analysis of the Oxford sequence types (STs) revealed distinct patterns across various states/regions in Nigeria. For instance, in Adamawa state, Northeast region, a limited diversity of STs was observed, with two types identified, of which ST2476 was the most prevalent, occurring in three *A. baumannii* isolates. In the Kano state Northwest region, three STs were detected, with ST942 found to be mostly prevalent, occurring in 6 *A. baumannii* isolates, while ST1051 and ST2832 occurred in one isolate each. Osun State, South-west Nigeria, displayed a more extensive range of STs, with 11 distinct types identified in Osun State, with ST1089 detected in eight *A. baumannii* isolates, followed by ST862 (three isolates), ST229 (two isolates), and single occurrences of other STs. Also, in another South-west region, Ibadan, Oyo state, a significantly higher diversity of STs was observed, with 22 distinct types recorded. However, ST472 was the most ST detected in seven *A. baumannii* isolates, followed by ST28 (six isolates) and ST2151 (five isolates). Notably, among isolates with undefined sources, 19 distinct STs were documented, with two types, ST229 and ST231, emerging as the most frequent, each occurring five times, followed by ST24283, which was found in four isolates. However, a high proportion of the sequences of unknown Oxford ST (*n* = 62) were successfully assigned to an ST using the Pasteur scheme.

**Fig. 3:**
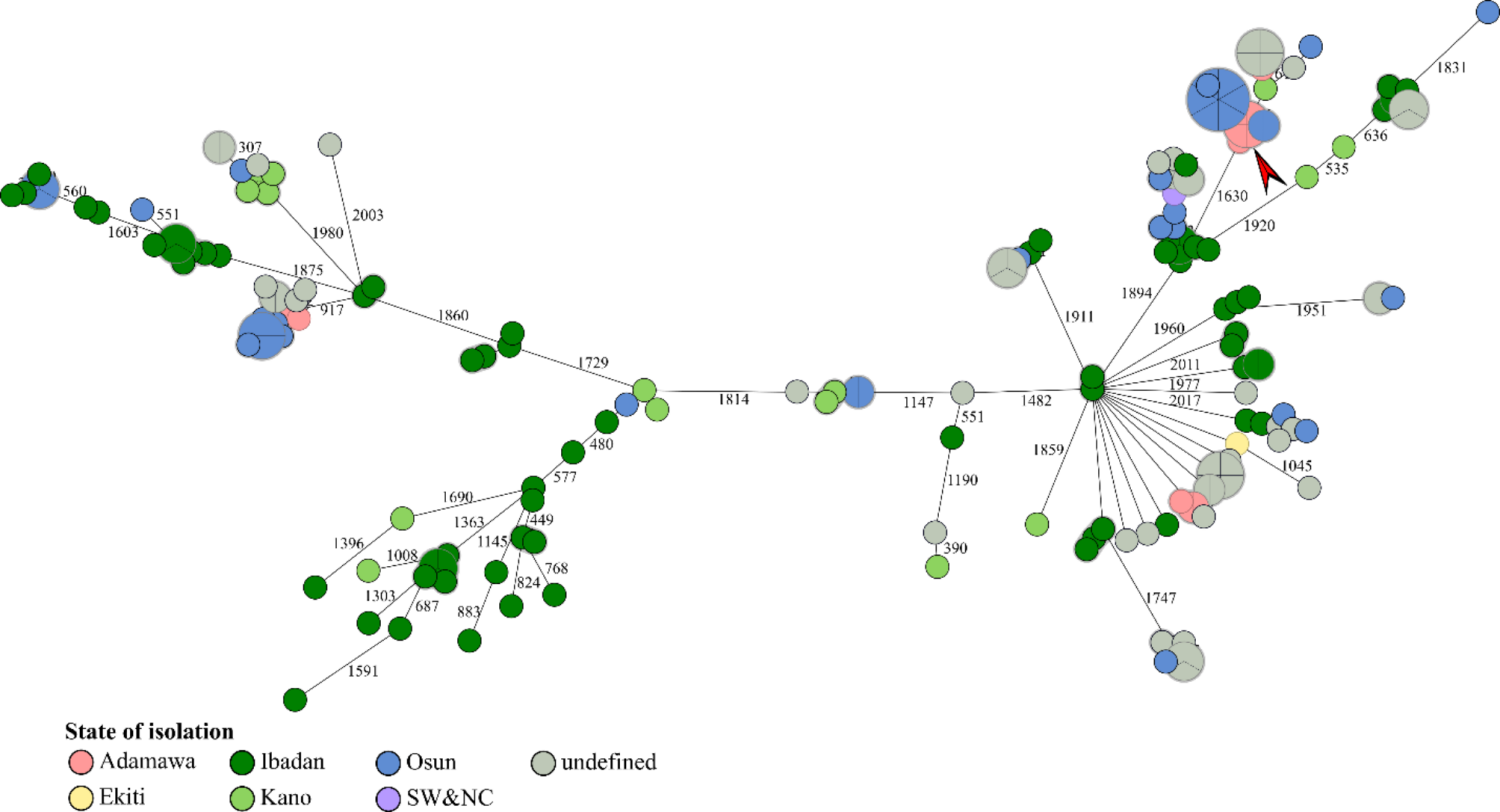
Minimum spanning tree based on cgMLST allelic differences of the Nigerian *A. baumannii* deposited in online repositories. Numbers on branches indicate allelic differences. The red arrow indicates a cluster of samples from various sources sharing the same cgMLST profile

Based on the cgMLST allelic distances, a minimum spanning tree was created (Fig. 3**)** that revealed large differences between Nigerian *A. baumannii* isolates regarding their cgMLST profiles, indicating the presence of multiple distinct lineages. Notably, allelic differences between isolates were quite high (mostly > 2000 alleles). In particular, isolates from Ibadan were highly diverse in their cgMLST profile, but isolates from other states were found on different branches of the tree. Only a few clusters were observed that shared similar allelic profiles, indicating potential epidemiological links. If there were identical profiles, the isolates mostly originated from the same state and source. There was one markable exception, where samples from farmland, a school toilet floor, a door handle, and a hospital bed were identical in their cgMLST profile (marked by a red arrow). These isolates all came from Adamawa.

### In-silico detection of antibiotic resistance genes in *A. baumannii* strains

The in-silico analysis of antimicrobial resistance (AMR) determinants identified 168 distinct AMR genes across 12 different antibiotic classes. Additionally, the presence of multiple efflux systems was noted, underscoring the significant diversity of resistance mechanisms in *A. baumannii* isolates from Nigeria deposited in public repositories. With the ResFinder database, 168 antimicrobial genes were specifically developed to identify acquired resistance genes from raw sequence data [34], and 86 of the 168 AMR genes were successfully identified. However, the *ant* (3″)-*IIa*, *bla*_VIM_, and most members of the *bla*_ADC_ gene family were not detected with this database. In contrast, the usage of the Comprehensive Antibiotic Resistance Database (CARD) and the National Center for Biotechnology Information (NCBI) databases effectively identified these non-β-lactamase genes within the available whole-genome sequencing (WGS) data.

The most prevalent resistance genes were those conferring resistance to cephalosporin antibiotics, with 43 distinct gene variants identified. Among these, 41 variants of the *bla*_ADC_ gene were observed, with *bla*_ADC−79_ emerging as the most frequent, detected in 45 (23.81%) isolates. Other prominent *bla*_ADC_ variants included *bla*_ADC−2_ (13.76%), *bla*_ADC−30_ (11.11%), and several variants found in at least one isolate each (Table 1).

**Table 1:** The frequency of distribution of AMR genes and mechanism of resistance in identified deposited 189 *A. baumannii* from Nigeria in public repositories

Antibiotic Class (F)	Antibiotics	AMR gene (F)	Mechanism
Aminoglycosides (21)	Aminoglycosides	*mgrA* (1)	Modification of ribosomal target
		*aacA16* (4)	Modification of antibiotics
	Gentamycin	*aac(6’)-Ib4* (1), *aac(3’)-IId* (19), *aac(3’)-IIe* (5)	
		*armA* (15)	Methylation of the 16 S rRNA target
		*aac(3’)-Ia* (38)	Antibiotics inactivation
	Amikacin/kanamycin/dibekacin	*aac(6’)-Iaf* (4)	Antibiotics inactivation
	Amikacin/kanamycin/tobramycin	*aac(6’)-Ian* (4), *aac(6’)-Ib9* (2)	
	Gentamicin/kanamycin/tobramycin	*ant(2’)-Ia* (55)	
	Spectinomycin/streptomycin	*ant(3’)-IIa* (31)	
	Streptomycin	*ant(3’)-Ia* (31), *aph.(3’)-Ib* (79), *aph.(6’)-Id* (79)	
	Kanamycin	*aph(3’)-Ia* (49)	
	Amikacin/kanamycin	*aph.(3’)-vI* (29), *aph.(3’)-vIa* (19), *aph.3…vIb* (2)	
	Streptomycin	*aadA1* (42), *aadA2* (2)	Antibiotics modification
Phosphonic antibiotics (3)	Fosfomycin	*abaF* (147)	Efflux pump production
		*fos* (27)	Antibiotics inactivation
		*fosB* (1)	
Quinolone (6)	Fluoroquinolone	*AbaQ* (173), *norA* (1)	Efflux pump production
	Quinolone	*gyrA: S81L* (120), *parC.E88K* (1), *parC.S84F* (7), *parC.S84L* (104)	Target modification
Streptothricin (2)	Streptothricin	*sat-1* (9), *sat-2* (10)	Antibiotics modification
Phenicol (5)	Chloramphenicol	*catI* (3), *catA1* (3)	Antibiotics modification
		*cmlA5* (15), *cmlB1* (16)	Efflux pump production
	Chloramphenicol/florfenicol	*floR* (14)	
Bleomycin (1)	Bleomycin	*ble*5 (51)	Drug sequestration
Trimethoprim (6)	Trimethoprim	*dfrA1* (12), *dfrA20*, (13) *dfrC* (2), *dfrA44* (10), *dfrA45* (1), *dfrS1* (1)	Target modification
Macrolide (5)	Lincosamide	*lnuA* (1)	Antibiotics modification
	Erythromycin	*mphE* (55), *msrE* (56)	Antibiotics modification
	Rifamycin	*arr-2* (15), *arr.3* (2)	Antibiotics modification
Sulfonamide (2)	Sulfonamide	*sul1* (55), *sul2* (112)	Mutation of target site
Polymyxin E (1)	Colistin	*pmrB.T2321* (3)	Target modification
Tetracycline (5)	Tetracycline	*tet*.*39* (37), *tet.G* (2), *Tet.X3* (2).	Efflux pump production
		t*et(A)* (2), *tet(B)* (47)	Blocking of the target site
Cephalosporin (43)	Cephalosporin	*bla*_*ADC-10*_ (10), *bla*_*adc.11*_ (6), *bla*_*adc.2*_ (26), *bla*_*adc.25*_ (1), *bla*_*adc.26*_ (15), *bla*_*adc.3*_ (15), *bla*_*adc.30*_ (21), *bla*_*adc.5*_ (8), *bla*_*adc.32*_ (12), *bla*_*adc.50*_ (3), *bla*_*adc.52*_ (5), *bla*_*adc.58*_ (14), *bla*_*adc.6*_ (10), *bla*_*adc.75*_ (1), *bla*_*adc.76*_ (9), *bla*_*adc.78*_ (10), *bla*_*adc.79*_ (45), *bla*_*adc.80*_ (2), *bla*_*adc.87*_ (12), *bla*_*adc.99*_ (5), *bla*_*adc.154*_ (8), *bla*_*adc.158*_ (4), *bla*_*adc.163*_ (4), *bla*_*adc.164*_ (1), *bla*_*adc.165*_ (7), *bla*_*adc.169*_ (3), *bla*_*adc:176*_ (2), *bla*_*adc.191*_ (7), *bla*_*adc.196*_ (1), *bla*_*adc.199*_ (2), *bla*_*adc.203*_ (3), *bla*_*adc.238*_ (2), *bla*_*adc.248*_ (3), *bla*_*adc.258*_ (1), *bla*_*adc.269*_ (2), *bla*_*adc.279*_ (2), *bla*_*adc.291*_ (10), *bla*_*adc.318*_ (1), *bla*_*adc.321*_ (1), *bla*_*adc.341*_ (1), *ampc_beta.lactamase*, (8), *bla*_*per.7*_ (7)	Ambler class C beta-lactamase
	Cephalosporin/penicillin	*tem.*12 (16)	Antibiotic inactivation
Carbapenem (48)	Carbapenem	*tem:1* (16)	Antibiotic inactivation
		*blal* (1), *pc1_beta.lactamase.blaz* (1), *carb.16* (9), *carb.2* (1), *carb.3* (2), *blamca* (8), *blar1* (1), *blarsa.1* (1), *ndm.1* (50), *ndm.6* (1), *vim.5* (3), *oxa.100* (9), *oxa.106* (2), *bla*_*oxa.120*_ (3), *bla*_*oxa.121*_ (4), *bla*_*oxa.122*_ (4), *bla*_*oxa.144*_ (1), *bla*_*oxa.180*_ (10), *bla*_*oxa.203*_ (1), *bla*_*oxa.208*_ (3), *bla*_*oxa.217*_ (58), *bla*_*oxa.23*_ (2), *bla*_*oxa.337*_ (1), *bla*_*oxa.338*_ (7), *bla*_*oxa.343*_ (2), *bla*_*oxa.374*_ (6), *bla*_*oxa.378*_ (3), *bla*_*oxa.383*_ (1), *bla*_*oxa.402*_ (9), *bla*_*oxa.411*_ (4), *bla*_*oxa.420*_ (9), *bla*_*oxa.429*_ (16), *bla*_*oxa.441*_ (4), *bla*_*oxa.51*_ (9), *bla*_*oxa.58*_ (16), *bla*_*oxa.64*_ (13), *bla*_*oxa.66*_ (22), *bla*_*oxa.67*_ (1), *bla*_*oxa.68*_ (9), *bla*_*oxa.69*_ (19), *bla*_*oxa.70*_ (3), *bla*_*oxa.78*_ (1), *bla*_*oxa.88*_ (3), *bla*_*oxa.90*_ (1), *bla*_*oxa.91*_ (9), *bla*_*oxa.94*_ (17), *bla*_*oxa.98*_ (4).	Ambler class D beta-lactamase

*F in parenthesis equals “number of occurrence of AMR genes from189 total number of isolates

Carbapenem resistance was encoded by 48 distinct AMR genes, with 38 genes belonging to the oxacillinase encoding genes, wherein *bla*_OXA−23_ was the most common variant, detected in 58 (30.69%) isolates, followed by *bla*_OXA−66_ (11.64%). Among the Ambler class B beta-lactamase encoding genes, *bla*_NDM−1_ was the most frequently encountered, present in 50 (26.46%) isolates (Table 1). Twenty-one AMR genes conferring resistance to aminoglycosides were identified. Genes *aph (3”)-Ib* and *aph(6)-Id*, conferring resistance to streptomycin, were the most prevalent, detected in 79 (41.79%) genomes (Table 1). Six genes conferring resistance to quinolones were identified, with *abaQ* being the most frequent, detected in 173 (91.53%) strains. Further, several point mutations that might confer resistance were detected: *gyrA*: S81L (63.49%), *parC*.S84L (53.03%), and *parC.*E84F (3.70%).

Seventeen multi-efflux system genes conferring resistance to multiple antibiotic classes were identified. Interestingly, *abeS*,* adel*,* adeL*, and *adeM* were the most prevalent, detected in all (100%) strains. Other genes encoding for the multi-efflux system were present in at least 147 (77.78%) genomes. Furthermore, other AMR genes, including three fosfomycin resistance encoding genes, were identified, with *abaF* being the most prevalent (77.78%) (Table 2).

**Table 2:** The frequency of distribution of multiple efflux system and mode of action in identified deposited 189 *A. baumannii* from Nigeria in public repositories

Resistance mechanism	Antibiotics	AMR genes	Mode of action
Multi-Efflux (17)	Multi-drug	*amvA* (186)	Efflux pump production
		*abeS* (189), *RSA.1* (1)	Enzyme regulator for antibiotic inactivation
		*adeC* (57), *adeA* (147), *adeB* (159), *adeI* (189), *adeJ* (188), *adeK* (188), *adeL* (189), *adeN* (179), *adeR* (148), *adeS* (151)	Antibiotics inactivation
	Fluoroquinolone, tetracycline	*adeF* (187), *adeG* (187), *adeH* (183)	Antibiotics inactivation
	Mdr- fluoroquinolone, tetracycline, colistin	*abe*M (189)	Antibiotics inactivation

*F in parenthesis equals “number of occurrence of AMR genes; *189 is the total number of isolates

Notably, all the isolates investigated harbored AMR genes encoding for two or more antibiotic classes, except for isolates with the accession numbers ERR10782663, ERR10852925, ERR10852932, ERR10852996, ERR10854476, and ERR6938153, which harbored AMR genes against just one antibiotic class. Five of 189 *A. baumannii* genomes harbored several genes encoding for eleven different antibiotic classes, while ten strains harbored different genes responsible for resistance against ten antibiotic classes.

**Fig. 4:**
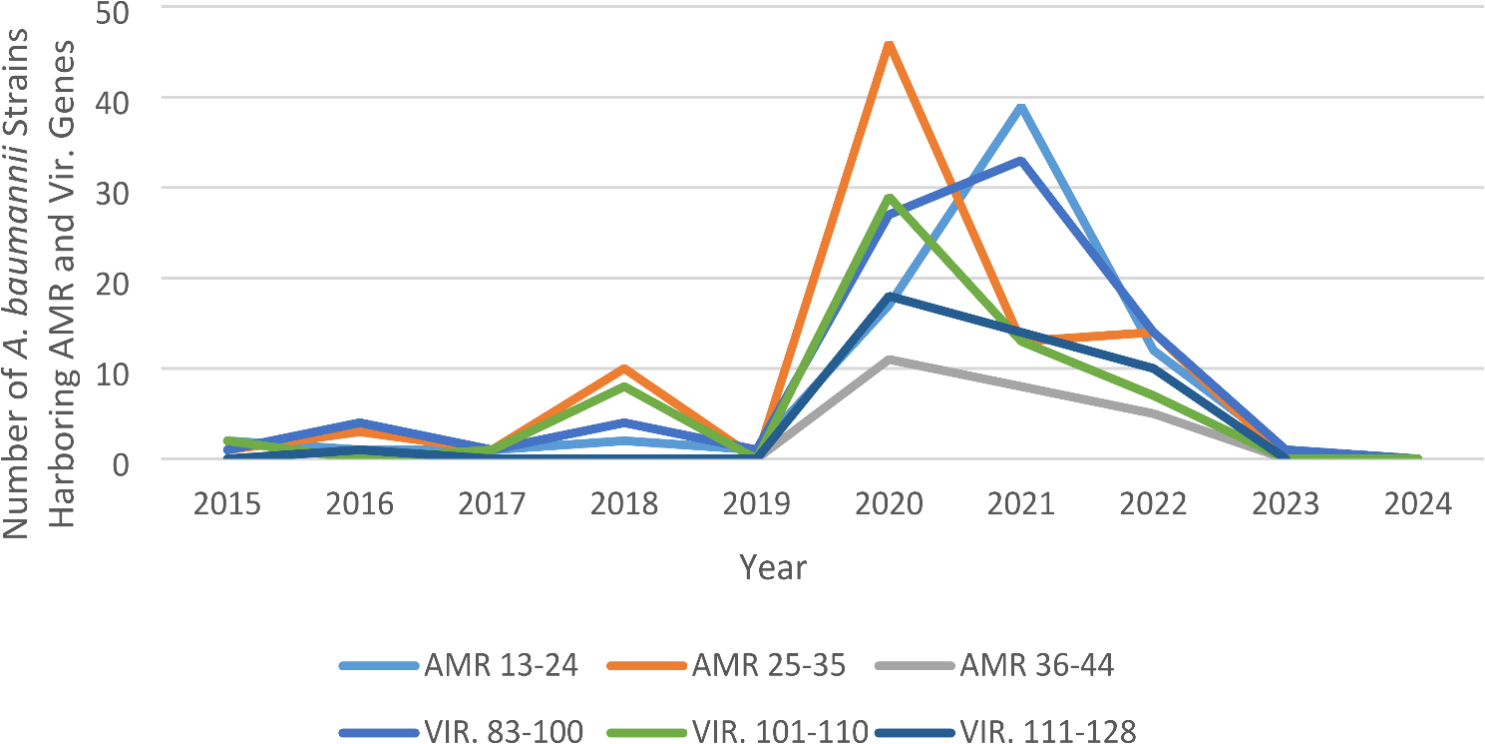
The variation in the number of AMR and virulence genes (in ranges) among 189 *A. baumannii* isolates deposited in public repositories between 2015 and 2024

The variation observed from AMR results in 189 *A. baumannii* isolates deposited in public repositories between 2015 and 2024 is shown in Fig. 4. The analysis of this result indicated that between 13 and 44 AMR genes were harbored by at least one of the 189 *A. baumannii* strains. The number of these AMR genes was grouped into three ranges: 13–24, 25–34, and 35–44. Generally, the number of isolates that harbored a higher number of AMR genes occurred between 2020 and 2021. The number of *A. baumannii* isolates harboring AMR genes ranged from 13 to 24, which was 2 in 2015 but rose to 17 in 2020 and peaked in 2021 with 39 isolates. However, this declined to 1 isolate in 2023. Similarly, only one isolate harbored the range of 25–35 AMR genes in 2015, but it increased to 10 in 2018, peaked at 46 isolates in 2020, and declined to 13 isolates in 2021. Furthermore, 11 *A. baumannii* isolates were found to harbor AMR genes in the range 36–44 in 2020. But declined to 5 in 2022.

### In-silico detection of virulence genes in *A. baumannii* strains

The virulence genes analysis revealed a total of 137 genes encoding six distinct virulence mechanism groups in the *A. baumannii*. Notably, most of the genes (*n* = 38) could be attributed to nutritional/metabolism factors, followed by 32 genes involved in effector delivery systems, 28 genes associated with adherence, 20 genes related to immune modulation, 18 genes encoding for biofilm production, and the smallest group (3 genes) responsible for enterotoxin production. Interestingly, a ubiquitous presence of specific genes was observed across the entire cohort of *A. baumannii* samples, with 100% of the strains harboring the *basj* gene, which is involved in the biosynthesis of acinetobactin. Moreover, the genes *ipsB*,* ipxS*,* ipxC*, and *ipxD*, involved in immune modulation, were uniformly present in all genome sequences, as were the *plC* gene, encoding for enterotoxin production, and seven genes (*gspC*,* gspD*,* gspF*,* gspH*,* gspL*,* gspM*, and *gspN*) responsible for effector delivery systems. Furthermore, a majority (187 strains, or 98.97%) of the strains possessed the *adeF* and *adeG* genes, which are involved in biofilm production (Table 3**)**. The analysis of differences in the number of virulence genes harbored in 189 *A. baumannii* isolates is shown in Fig. 4. The result indicated that between 83 and 128 virulence genes were harbored by at least one of the 189 *A. baumannii* strains. The number of these virulence genes was grouped into three ranges: 83–100, 100–110, and 111–128. Generally, the number of isolates that harbored a higher number of virulence genes occurred between 2020 and 2021. The number of the *A. baumannii* isolates harboring virulence genes range of 83–100 was 1 in 2015 but rose to 27 in 2020 and peaked in 2021 with 33 isolates recorded and declined to 1 isolate in 2023. Similarly, two isolates harbored the range of 100–110 virulence genes in 2015 but increased to 8 in 2018, peaked at 29 isolates in 2020, and then declined to 7 isolates in 2022. Furthermore, the number of *A. baumannii* isolates harboring the range 111–128 virulence genes peaked in 2020, with 18 isolates obtained but declined to 10 in 2022.

**Table 3:** The mechanism and frequency of virulence genes detected in investigated *A. baumannii*

Category	Virulence genes (Frequency)
Nutrition\Metabolic Factor (38)	ACICU_RS00400 (143), ACICU_RS00405 (143), ACICU_RS00445 (4), ACICU_RS00450 (5), ACICU_RS004550 (7), ACICU_RS00460 (1), ACICU_RS00465 (1), ACICU_RS004700 (22), ACICU_RS00475 (69), ACICU_RS004100 (187), ACICU_RS00500 (188), ACICU_RS04565 (161), ACICU_RS04570 (61), ACICU_RS04575 (61), ACICU_RS045100 (59), ACICU_RS045100 (61), ACICU_RS045100 (60), ACICU_RS045100 (61), ACICU_RS04605 (61), ACICU_RS04610 (167), *barA* (186), *barB* (187), *basA* (181), *basB* (183), *basC* (183), *basD* (184), *basF* (185), *basG* (187), *bash* (188), *basI* (184), *basJ* (189), *bauA* (31), *bauB* (183), *bauC* (179), *bauD* (178), *bauE* (179), *bauF* (184), *entE* (185)
Immune Modulation (20)	ACICU_RS003100 (143), *cpaA* (58), *gale* (169), *galU* (184), *lpsB* (189), *lpxA* (189), *lpxB* (189), *lpxC* (189), *lpxD* (189), *lpxL* (188), *lpxM* (188), *omp*A (188), *pbpG* (189), *pgi* (189), *pseB* (17), *pseC* (17), *pseF* (16), *pseG* (15), *pseH* (16), *pseI* (17)
Adherence (28)	*ata* (24), ACICU_RS16100 (180), *fimT* (189), *fimU* (180), *fimV* (188), *gspO0pilD* (189), *pilA* (31), *pilB* (188), *pilC* (188), *pilE* (180), *pilF* (188), *pilG* (189), *pilH* (189), *pilI* (188), *pilJ* (187), *pilM* (189), *pilN* (189), *pilO* (189), *pilP* (189), *pilQ* (189), *pilR* (189), *pilS* (186), *pilT* (188), *pilU* (188), *pilV* (185), *pilX* (179), *pilY1* (160)
Biofilm (16)	*csuA* (150), *csuA0B* (157), *csuB* (146), *csuC* (165), *csuD* (174), *csuE* (167), *pgaA* (183), *pgaB* (181), *pgaC* (178), *pgaD* (179), *abaI* (149), *abaR* (145), *adeF* (187), *adeG* (187), *adeH* (183), *bap* (54)
Enterotoxin (3)	*plc1* (182), *plc2* (186), *plcD* (189)
Effector delivery system (32)	*tagX* (128), *tsaP* (188), *tse1* (13), *tse2* (10), *tse4* (1), *tssA* (104), *tssB* (92), *tssC* (91), *tssE* (92), *tssF* (90), *tssG* (99), *tssK* (105), *tssL* (120), *tssM* (100), *tviB* (115), *tgrG0tssI* (155), *clpV0tssH* (105), *esxA* (1), *gspC* (189), *gspD* (189), *ngspE1* (186), *gspE2* (188), *gspF* (189), *gspG* (179), *gspH* (189), *gspI* (189), *gspK* (188), *gspL* (189), *gspM* (189), gspN (189), *hcp0tssD* (92), *hemO* (61)

*F in parenthesis equals “number of occurrence of AMR genes; *189 is the total number of isolates

## Discussion

The remarkable resilience of *A. baumannii* in the face of adverse environmental conditions, coupled with its propensity to develop or acquire resistance, renders it a paramount nosocomial pathogen [1]. *A. baumannii* is a One-Health pathogen, difficult-to-treat, and its accurate diagnosis is challenging [3, 35]. Elucidating the AMR genes circulating in *A. baumannii* is crucial for understanding the underlying mechanisms driving the acquisition and development of antimicrobial resistance [36]. This study, therefore, investigated the diversity, antimicrobial resistance genes, and virulence genes in 189 publicly available *A. baumannii* genomes from various laboratories across Nigeria.

Literature on the prevalence of sequence types (STs) of *A. baumannii* in Nigeria is limited. To date, only a small number of STs from human-derived *A. baumannii* isolates have been documented [37, 38]. The cgMLST analysis revealed a complex community, indicating the existence of distinct lineages, epidemiological links between isolates, and the presence of genetically diverse isolates that may have originated from different sources. This finding is consistent with previous studies that have reported the presence of diverse clonal complexes in *A. baumannii* populations from other countries [39, 40].

The Pasteur MLST scheme identified 49 distinct sequence types (STs) among 181 out of 189 strains, with a typing efficiency of 95.77%. This is comparable to previous studies that have reported typing efficiencies ranging from 90 to 95% using the Pasteur scheme [39, 41]. ST2 emerged as the most frequent across all *A. baumannii* whole genome sequences. This corresponds with previous studies that have reported ST2 as a common and widespread ST in *A. baumannii* populations globally [39, 42, 43]. It is well established that ST2 is affiliated with the international clone II (IC2), which has been reported to be the most prevalent type worldwide [20]. The study recorded ST2 (an IC2) in 22 *A. baumannii* isolates from different locations in Nigeria: Osun South-West (10 strains), Adamawa North-East (6 strains), Kano Northwest (1 strain), and an additional 5 from unspecified locations. This geographical spread of this ST raises concerns about the potential for regional outbreaks and the challenges they pose to healthcare systems. This is because it was observed in some non-hospital environments as some of the ST2 strains were documented in the public repositories to have been recovered from the hospital door handles, hospital beds, hospital drawers, school toilet floors, unpolluted fields, and untreated hospital wastewater. The presence of these strains indicates that *A. baumannii*, particularly the MDR variants, is not confined to a specific locality but a nationwide concern, thus requiring a coordinated response to manage its spread. Furthermore, this study recorded widespread distribution of 15 ST85 *A. baumannii* strains belonging to IC9 in Nigeria, particularly in Osun (South-West), Adamawa (North-East), and some undefined locations. ST85/IC9 is a high-risk lineage linked to MDR and global outbreaks due to its propensity to acquire carbapenemase genes such as *bla*_NDM−1_ and *bla*_OXA−23_ [44]. This strain of *A. baumannii* was found to harbor high number of AMR and virulence genes in this study, hence, poses a public health threat. Its presence in geographically dispersed regions of Nigeria highlights endemic circulation and underscores the urgent need for genomic surveillance, as IC9 strains are often associated with prolonged hospital outbreaks and horizontal gene transfer of resistance determinants via mobile genetic elements like Tn125 and Tn2006 transposons [13, 15]. The identification of these strains in undefined sources and school toilet floors (environmental samples) further signals ecological persistence and potential community transmission routes, particularly given this pathogen’s ability to survive desiccation and form biofilms [45]. In North Africa, such as Sudan [17] and other regions globally, including Belgium (Europe), Italy (Europe), Egypt (North Africa), and Pakistan (Middle East) [21, 45–46], IC9 strains have been reported, and suggestions on the need to re-evaluate infection prevention protocols and strengthen molecular surveillance infrastructure in these countries have been advocated. Therefore, tracking IC9 dissemination is imperative to inform region-specific containment strategies and antibiotic stewardship programs in Nigeria. In contrast, the Oxford MLST scheme assigned 62.96% of the isolates to STs, with 44 distinct STs identified among 119 strains. This agrees with the findings of Wareth et al. [39], who reported lower typing efficiencies using the Oxford scheme. The comparison of the two MLST schemes reveals that the Pasteur scheme was more convenient in typing *A. baumannii* strains, and therefore, more defined STs are known than the Oxford scheme [39, 41]. The findings are consistent with previous studies and highlight the importance of using reliable and efficient typing schemes, such as the Pasteur scheme, for epidemiological surveillance and outbreak investigations [39–41].

Furthermore, 168 AMR genes encoding for 12 different antibiotic classes within the *A. baumannii genome* were recorded. It was observed that 22 *A. baumannii* strains belonging to ST2 harbored a range of 31 to 44 AMR genes. Specifically, 59% (13/22) were found to possess 35 AMR genes, and 31.82% (7/22) with 31 to 33 AMR genes. Interestingly, two *A. baumannii* strains with accession codes ERR4783188 and ERR6938099 harbored 44 and 41 AMR genes, respectively. Similarly, all *A. baumannii* ST85 harbored between 26 and 31 AMR genes. Within this subset, 46.67% (7/15) of the strains possessed 27 distinct AMR genes, while 13.33% (2/15) harbored 28 AMR genes, and so forth. Interestingly, of the three AMR databases used in this study, the ResFinder database documented 86 out of 168 AMR genes that were successfully detected, but key genes like *ant* (3″)-IIa and *bla*_VIM_, as well as many *bla*_ADC_ family genes, were missed. In contrast, using CARD and NCBI databases, these AMR genes could be detected in the deposited WGS data, underscoring the importance of utilizing comprehensive databases for accurate AMR detection. Therefore, the critical need for a multi-database approach when characterizing the in-silico resistance profiles of *A. baumannii* isolates is obvious [16].

The *Acinetobacter*-derived cephalosporinase (ADC)-type class C beta-lactamases are cephalosporinases with extended-spectrum resistance to cephalosporins [46]. These genes were the most prevalent resistance genes, with 41 distinct gene variants identified. The *bla*_ADC−79_ was the most frequent and has also been documented in clinical *A. baumannii* isolates in Groningen (The Netherlands) [47] and in Tunisia [48]. The low prevalence of *bla*_ADC−25−like_, *bla*_ADC−75_, and *bla*_ADC−87_ genes in Nigerian *A. baumannii* might be due to the nature of antibiotics that are usually prescribed to patients in Nigerian hospitals, limited use of cephalosporin antibiotics due to high cost and less abuse of this class of antibiotics [49].

Also, this study identified 48 distinct carbapenemase-encoding genes, with 38 belonging to the *bla*_OXA−51−like_ carbapenemase gene family. The *bla*_OXA−23_ gene was the most prevalent variant in this group, detected in 58 (30.69%) isolates, followed by *bla*_OXA−66_ (11.64%). Both belong to the Ambler class D beta-lactamases, which were originally considered relatively rare. The OXA beta-lactamase group genes, including *bla*_*OXA−23*_ and *bla*_*OXA−51*_, are known to spread on plasmids, facilitating transmission between bacterial species [50]. Several studies have demonstrated the association of these genes with resistance to all beta-lactam antibiotics, including carbapenems in Nigeria [51–55]. The *bla*_OXA−66/OXA−51−like_ carbapenemase contributes to intrinsic resistance to imipenem in clinical *A. baumannii* strains [51, 55]. The *bla*_OXA−51_ gene, first identified in *A. baumannii* from Argentina in 1996, represents the largest group of intrinsic OXA-type beta-lactamase genes and serves as an important marker for *A. baumannii* species identification [55–59]. The oxacillinase *bla*_OXA−23_, initially identified in 1993 in *A. baumannii* strains from the United Kingdom, has since been widely detected and linked to the global spread of carbapenem-resistant *A. baumannii* [60].

Additionally, 21 AMR genes conferring resistance to aminoglycosides were identified. The most common genes identified, *aph (3”)-Ib* and *aph (6)-Id*, potentially confer resistance to streptomycin and were detected in approximately 42% of the genomes. This is consistent with previous studies that have reported the widespread presence of aminoglycoside resistance genes in bacterial pathogens [61, 62]. The identification of six quinolone resistance genes, with *aba*Q being the most frequent, highlights the widespread use of quinolones in clinical practice, suggesting that quinolone resistance is a large concern in Nigeria [63]. Various multi-efflux system genes that confer resistance to multiple antibiotic classes in *A. baumannii were* identified. The most prevalent genes, *abe*S, *adeL*, and *adeM*, were detected in 100% of strains.

Most *A. baumannii* isolates are intrinsically resistant to chloramphenicol, although the mechanism responsible for this resistance remains unclear [64]. Five AMR genes conferring resistance to tetracycline compounds were identified, with *tet*(B) being the most prevalent (24.87%). The *tet*(B) gene encodes a tetracycline efflux protein that confers tetracycline resistance but not tigecycline resistance [65]. In contrast, *tet*(A) encodes for tigecycline resistance, which is a glycylcycline developed to overcome tetracycline resistance in *A. baumannii* [66]. The *tet*(A)6 gene was present in only two genomes. Two AMR genes conferring resistance to sulfonamides were identified, with *sul*2 being the most prevalent. Both genes are mediated by transposons and plasmids and express dihydropteroate synthases in Gram-negative bacteria, conferring resistance to sulfonamides [8]. Additionally, five genes encoding trimethoprim resistance were found, with *dfrA20* and *dfrA1* being the most frequent. The presence of one or both genes in *A. baumannii* isolates could confer resistance to trimethoprim/sulfamethoxazole.

Colistin resistance in *A. baumannii* is linked to mutations in PmrAB [67]. Interestingly, no plasmid-mediated colistin resistance (*mcr* genes) was detected. Other resistance genes identified included *sat-1* (4.76%) and *sat-2* (5.29%) (both encode resistance to streptothricin), *ble* (encodes resistance to bleomycin, 26.96%), and *inu*A (encodes resistance to lincosamide, 0.53%).

Notably, aside from possessing at least 11 genes encoding for multi-efflux systems, all the isolates’ genomes contained AMR genes that could confer resistance to two or more antibiotic classes. Among the 189 *A. baumannii* strains analyzed, five exhibited genes potentially conferring resistance to 11 distinct antibiotic classes, whereas ten strains possessed genes responsible for resistance to ten different antibiotic classes. This implies that *A. baumannii* strains circulating within the clinical and environmental setting in Nigeria harbor the potential of being MDR and extensively drug-resistant (XDR), with the likelihood of pan-drug resistant (PDR) strains emerging. It is noteworthy that the substantial burden of AMR genes in both *A. baumannii* strains ST2 and ST85 highlights an urgent need for enhanced genomic surveillance and collaborative efforts across sectors to combat the rising threat of antimicrobial resistance. By adopting a One-Health perspective, stakeholders can develop more effective strategies for monitoring and controlling resistant pathogens, ultimately protecting public health and ensuring effective treatment options remain available [68].

All ST2 isolates contained between 94 and 110 distinct virulence genes, with two exceptions exhibiting even higher counts—126 and 115 genes, respectively. Similarly, ST85 strains showed a range of 98 to 109 virulence genes, with one isolate (accession code ERR4783222) possessing 114 genes. The identified virulence genes are implicated in various mechanisms that enhance the pathogenicity of *A. baumannii*. These include nutritional and metabolic functions critical for survival in hostile environments; effector delivery systems that facilitate infection processes; biofilm formation, which aids in persistence on surfaces; adhesion to host tissues; immune modulation to evade host defenses; and enterotoxin production that can disrupt host physiological functions [69]. This genetic variability underscores the adaptability and potential threat posed by these strains in clinical and non-clinical settings [69, 70]. Specifically, 38 virulence genes related to nutritional/metabolism factors were identified, with the *basj* gene being ubiquitous across all strains, highlighting the importance of metabolic adaptability in *A. baumannii* for survival in diverse environments [71]. Also, the presence of 32 genes involved in effector delivery systems, including a consistent set of seven genes (*gspC*,* gspD*,* gspF*,* gspH*,* gspL*,* gspM*,* gspN*), aligns with findings from other studies that underscore the role of type II secretion systems in *A. baumannii* pathogenicity [72, 73]. The uniform presence of *ipsB*,* ipxS*,* ipxC*, and *ipxD* genes involved in immune modulation corroborated other studies that have identified immune evasion as a key strategy for *A. baumannii* systemic host invasion [74–76]. However, some studies report a more diverse set of immune modulation genes, indicating potential differences in immune evasion strategies among strains [77]. The high prevalence of 98.97% *adeF* and *adeG* genes involved in biofilm production underscores the importance of biofilms in *A. baumannii* virulence [78–80].

In this study, *A. baumannii* isolates harboring AMR and virulence genes occurred throughout the year of study except in 2024. Generally, the number of *A. baumannii* isolates that harbored a higher number of AMR and virulence genes occurred between 2020 and 2021. This study showed variability in the *A. baumannii* infection pattern in Nigeria. Consequently, the peak infection trend observed between 2020 and 2021 may not be unconnected to the lockdown imposed by the COVID-19 pandemic, resulting in an increased infection rate within this period due to the overwhelming health facilities that likely resulted in poor infection control measures at that time. This study from Nigeria presents the first comprehensive genomic analysis of all deposited *A. baumannii* isolates of Nigerian origin, uncovering a metadata input deficiency in public repositories. The findings reveal a high diversity of circulating strains. Furthermore, the study identified multiple strains of *A. baumannii* with various virulence genes and AMR genes encoding resistances to several antibiotic classes. Nevertheless, the lack of data on food animals or food products calls for the establishment of a continuous surveillance system for this pathogen. This study highlights the prevalence of antimicrobial resistance genes in *A. baumannii* in Nigeria, emphasizing the need for continued surveillance and monitoring of antimicrobial resistance patterns to inform effective treatment strategies and mitigate the spread of resistant strains.

## Conclusion

The geographical distribution reveals a predominance of strains from South-west Nigeria, highlighting regional variations that warrant further investigation. The high proportion of undefined sources among the sequenced strains emphasizes the need for improved surveillance and data collection efforts. Improved metadata curation is necessary for targeted research that is essential to elucidate the transmission pathways and reservoirs of *A. baumannii* in Nigeria. The MLST analysis emphasizes the importance of using multiple MLST schemes for comprehensive strain characterization. The prevalence of cephalosporin, carbapenem, and aminoglycoside resistance genes, as well as the presence of multi-efflux system genes, highlights the urgent need for effective infection control measures and antimicrobial stewardship programs. Overall, this study emphasizes the importance of continued genomic surveillance to inform public health policy, adherence to strict hygiene practices and infectious agent control strategies, and the implementation of antimicrobial stewardship to mitigate the spread of antimicrobial resistance in Nigeria and beyond.

## Electronic supplementary material

Below is the link to the electronic supplementary material.

Supplementary Material 1: **table 1**. Eligible *A. baumannii* Whole Genome Sequence Extracted Data. This supplementary file contains the raw data considered in the Data analysis, result sub-section. This entails a total of 189 genomes of Nigerian *A. baumannii* retrieved from NCBI (*n* = 28) and ENA (*n* = 161).

Supplementary Material 2: **table 2**. Multilocus Sequence Typing Pasteur Scheme of Eligible *A. baumannii* Whole Genome Sequence. This supplementary file is the raw data considered for the Multilocus Sequence Typing Pasteur Scheme of Eligible *A. baumannii* Whole Genome Sequence in the MLST analysis, result sub-section.

Supplementary Material 3: **table 3**. Multilocus Sequence Typing Oxford Scheme of Eligible *A. baumannii* Whole Genome Sequence. This supplementary file is the raw data considered for the Multilocus Sequence Typing Oxford Scheme of Eligible *A. baumannii* Whole Genome Sequence in the MLST analysis, result sub-section.

Supplementary Material 4: **table 4**. CgMLST of Eligible *A. baumannii* Whole Genome Sequence. This is the raw data used for the construction of the minimum spanning tree, as shown in Fig. 3.

Supplementary Material 5: **Table 5**. The Distribution of Antimicrobial Genes and their Phenotypes in the Eligible *A. baumannii* Whole Genome Sequence. This contains the raw data of AMR genes of Eligible *A. baumannii* Whole Genome Sequence used for Tables 1 and 2 in In-silico detection of antibiotic resistance genes in *A. baumannii* strains result, sub-section.

Supplementary Material 6: **table 6**. The Distribution of Virulence Genes, their Phenotypes in the Eligible *A. baumannii* Whole Genome Sequence. This contains the raw data of virulence genes of Eligible *A. baumannii* Whole Genome Sequence used for Table 3 in In-silico detection of virulence genes in *A. baumannii* strains result, sub-section.

## Data Availability

The datasets analyzed during the current study are available in the National Center for Biotechnology Information (NCBI) and European Nucleotide Archive (ENA) repositories. Accession numbers can be found in Supplement Table 1.
